# Phylogenetic Analysis and Pathogenicity of Avian Reoviruses Isolated from Viral Arthritis Cases in China 2010–2024

**DOI:** 10.3390/vetsci12040307

**Published:** 2025-03-28

**Authors:** Liping Liu, Xiao Lu, Xiaozhen Guo, Xiao Gong, Feng Hu, Yifei Jiang, Yuehua Gao, Xiuli Ma, Yufeng Li, Bing Huang, Zhuoming Qin, Minxun Song, Kexiang Yu

**Affiliations:** 1 Institute of Poultry Science, Shandong Academy of Agricultural Sciences, Jinan 250100, China; liuliping@saas.ac.cn (L.L.); luxi0515@163.com (X.L.);; 2 Qingdao Yibang Biological Engineering Co., Ltd., Qingdao 266000, China

**Keywords:** avian reovirus, viral arthritis and tenosynovitis, phylogenetic analysis, homology analysis, variant, pathogenicity

## Abstract

**Simple Summary:**

Avian reovirus (ARV) is an important pathogen affecting the global chicken industry. In recent years, the harm caused by ARV in Chinese chicken flocks has become increasingly severe. To determine the prevalence of ARV in China, we sequenced the specific sigma C (σC) genes of 69 ARV strains isolated from 14 provinces in China from 2010 to 2024, and found that these 69 strains could be divided into six clusters (I–VI), with VI cluster being the most common, accounting for 42.03%. Except for cluster V, each of the other five clusters could be divided into two subclusters. Homology analysis showed that ARV isolates in clusters II–VI had only 50.3 to 60.8% homology with the commercial S1133 vaccine strain which is derived from cluster I. The results of the pathogenicity test showed that the representative strains of the six different clusters all caused swelling of the footpads of SPF chickens. The present study demonstrated that most ARV strains currently prevalent in China have undergone significant genetic mutations compared to traditional vaccine strains.

**Abstract:**

Avian reovirus (ARV) is one of the main causes of viral arthritis, tenosynovitis, malabsorption syndrome (MAS), runting-stunting syndrome, and immunodepression. In recent years, due to the emergence of new ARV strains, outbreaks of the disease have brought significant economic losses to chicken flocks. To determine the prevalence of ARV in China from 2010 to 2024, a total of 409 tissue samples from different breeding farms were collected from chickens presenting clinical signs of lameness and swollen joints in various flocks located in 18 provinces. As performed on these tissue samples, the ARV-specific reverse transcription-polymerase chain reaction (RT-PCR) assay indicated 111 ARV-positive samples with a positive rate of 27.14%. After viral isolation from the necropsied chicken samples, 69 ARV strains were isolated, and specific sigma C (σC) genes were amplified and sequenced. The sequence analysis of σC genes showed that these 69 isolates were grouped into six clusters, including 14 ARV isolates from cluster I (20.29%), 12 ARV isolates from cluster II (17.39%), 3 ARV isolates from cluster III (4.35%), 8 ARV isolates from cluster IV (11.59%), 3 ARV isolates from cluster V (4.35%), and 29 ARV isolates from cluster VI (42.03%). Except for cluster V, each of the other five clusters could be divided into two subclusters. Homology analysis showed that ARV isolates in clusters II–VI had only 50.3 to 60.8% homology with the commercial S1133 vaccine strain which is derived from cluster I. The ARVs in subcluster Ia had high homology with the S1133 vaccine strain (93.5–98.0%), while the ARVs in subcluster Ib had a low homology with the S1133 strain (73.4–76.4%). Further, the cluster VI viruses, the main epidemic genotype in China, had only 50.3–55.7% homology with the S1133 strain. The results of the pathogenicity test showed that the representative strains of the six different clusters all caused swelling of the footpads in SPF chickens, and the incidence rate was not significantly different. The present study will be helpful in the understanding the prevalence of ARV strains in China and revealed the genetic differences between the ARV isolates and the commercial vaccine strain.

## 1. Introduction

Avian reovirus (ARV) belongs to the *Orthoreovirus* genus in the *Reoviridae* family, and it is a nonenveloped virus, 70–80 nm in diameter with an icosahedral structure. ARV contains ten double-stranded RNA (dsRNA) segments, including three size classes: Large (L1, L2, and L3), Medium (M1, M2, and M3), and Small (S1, S2, S3, and S4) [1]. Among them, the S1 gene encodes two nonstructural proteins (p10 and p17) and one structural protein (σC) which is involved in the cell attachment and production of ARV-specific neutralizing antibodies [2]. Due to the noticeable diversity of the σC gene, it has been used to classify ARVs into five or six clusters [3,4,5,6]. There has been a previous report classifying ARVs into seven clusters [7].

ARV spreads horizontally and vertically, infecting a wide range of birds worldwide [8]. Infected chickens present with malabsorption syndrome (MAS), runting-stunting syndrome, and immunosuppression, mainly in the form of viral arthritis and tenosynovitis [8,9]. Although the mortality of this disease is not high, it reduces growth performance and egg production. In addition, due to the immunosuppression caused by ARV, infected chickens are more susceptible to secondary infection, leading to severe economic losses in the poultry industry [10,11]. The continuous emergence of variant strains also makes the prevention and control of this disease increasingly difficult [12,13,14,15,16].

To prevent ARV infection and eliminate vertical transmission, four main strains (S1133, 1733, 2408, and 2177) have been used as live and inactivated vaccines to immunize poultry flocks worldwide. However, in recent years, there has been an increasing number of cases of viral arthritis and tenosynovitis-associated ARV in vaccinated flocks [17,18,19,20,21]. These results suggest that the commercial vaccine used at present cannot provide adequate protection against field challenges. Therefore, the present study investigated the molecular phylogeny of ARVs circulating in commercial flocks from 2010 to 2024 and analysed their sequence diversity. These findings will provide reliable evidence for future work identifying a new approach to the prevention and control of ARV infection.

## 2. Materials and Methods

### 2.1. Sample Collection

A total of 409 tissue samples (liver, intestine, and joint fluid mixtures from one or more chickens) were obtained from necropsied chickens with clinical signs of lameness and swollen joints in 409 different breeding farms located in 18 provinces of China. Each clinical tissue was diluted 5-fold with sterile phosphate-buffered saline (PBS, pH 7.4), homogenized, and subjected to three freeze–thaw cycles. After centrifugation for 15 min at 7000 rpm at 4 °C, the supernatant was aliquoted and stored for viral RNA extraction and virus isolation.

### 2.2. RNA Extraction and Diagnostic RT-PCR

A 200 µL sample of supernatant was used for RNA extraction using the AxyPrep^TM^ Body Fluid Viral DNA/RNA Miniprep Kit (AxyGen, San Francisco, CA, USA) in accordance with the manufacturer’s protocol. The purity and concentration of the extracted RNA were measured using a NanoDrop ONE (Thermo Fisher Scientific, Waltham, MA, USA), and the final product was stored at −80 °C. To determine the presence of the ARV genome, a diagnostic reverse transcription-polymerase chain reaction (RT-PCR) specific to ARV was performed to amplify a 284 bp ARV gene segment using a pair of diagnostic primers (F, 5′-TGCAGATGTGTCTYCAGTAYGT-3′; and R, 5′-CATRTCAGCCCATTCWG AAGG-3′).

### 2.3. Viral Isolation

The positive samples detected by diagnostic RT-PCR were subjected to virus isolation. After passing through a 0.22 µm filter, 0.1 mL of filtered supernatant was subsequently inoculated into the yolk sac of 7-day-old specific pathogen-free (SPF) chicken embryos. After 5 days, the allantoic fluids and infected embryos were harvested, homogenized, and centrifuged for subsequent propagation in the chicken embryos. After three blind passages, the harvested supernatant was detected by RT-PCR for ARV.

### 2.4. ARV Propagation in LMH Cells

In the present study, all ARV isolates were propagated using chicken liver hepatocellular carcinoma cells (LMH cells) cultured in Dulbecco’s Modified Eagle Medium (DMEM, Gibco, Shanghai, China) containing 10% foetal bovine serum (FBS, PAN, Adenbach, Germany). After viral adsorption at 37 °C for 1 h, cells were cultured in DMEM containing 1% FBS. When the cytopathic effect (CPE) reached 80%, cell cultures were subjected to two freeze–thaw cycles and stored at −80 °C.

### 2.5. Amplification, Sequencing, and Phylogenetic Analysis Based on the σC Gene

To amplify the σC gene of ARV, a pair of primers (SigmaC-F, 5′-TCAARYATTTGTGAGTACGATTG-3′; and SigmaC-R, 5′-GCCRCACCTTARGTGTC-3′) was designed to produce a fragment of 1100 bp. The RT-PCR product was confirmed by sequencing and cloned into the pMD18-T plasmid, which was then transformed into *Escherichia coli* DH5α cells. The positive clones were submitted to a commercial service for sequencing (Sangon Biotech, Shanghai, China).

The phylogenetic tree was constructed in MEGA7.0 using the neighbor-joining method with 1000 bootstrap replicates [22]. A homology analysis was performed using the ClustalW method in the MegAlign program using Lasergene 7.0 software.

### 2.6. Pathogenicity in SPF Chickens

Six different genotypes of ARV isolates were selected for pathogenic evaluation, named, respectively, the CZ18, PJ18, TJ22, BZ22, TS18, and WF17 strains. SPF chickens at 21 days of age were purchased and inoculated with 100 ELD_50_/0.1 mL of different ARV strains via the footpad. The control group was prepared and inoculated with saline solution. The chickens were kept in separate isolation units and provided with food and water ad libitum. The chickens were monitored and observed for the development of clinical signs.

## 3. Results

### 3.1. Diagnostic RT-PCR and Virus Isolation

**Figure 1:**
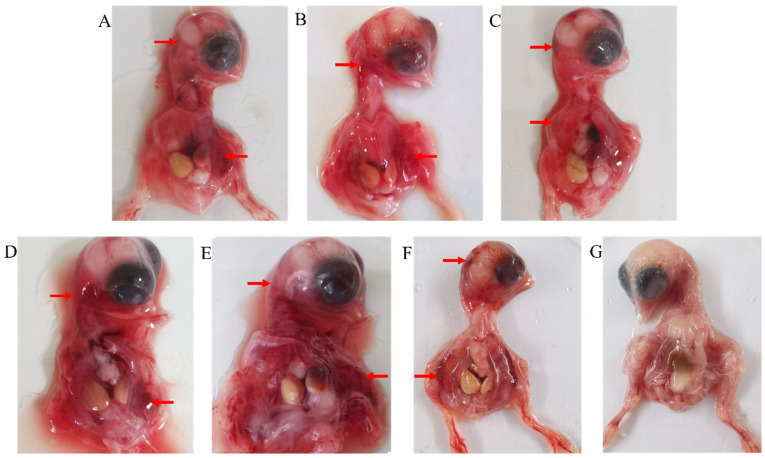
Pathological lesions of avian reovirus infections in SPF chicken embryos. (**A**–**F**) Chicken embryos were inoculated with different ARV isolates from clusters I–VI (CZ18, PJ18, TJ22, BZ22, TS18, and WF17). All of the embryos showed similar pathological lesions, such as hyperemia, hemorrhage, and edema. (**G**) A chicken embryo inoculated with PBS was used as the control.

Out of 409 tissue samples, diagnostic RT-PCR identified 111 positive samples with a positive rate of 27.14% (Table 1). These positive samples were processed and inoculated into the yolk sac of 7-day-old SPF chicken embryos. A total of 69 ARVs were isolated and identified by diagnostic RT-PCR, and all isolates caused the death of SPF chicken embryos. Infected SPF chicken embryos showed dysplasia, hyperemia, hemorrhage, edema, and liver necrosis (Figure 1).

### 3.2. ARV Propagation in LMH Cells

**Figure 2:**
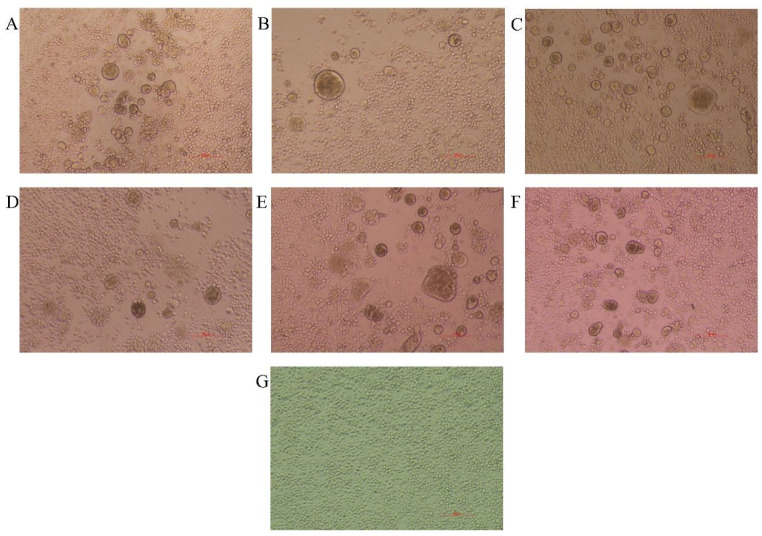
ARV isolate propagation in LMH cells. (**A**–**F**) Syncytia was observed in LMH monolayer cells infected with different ARV isolates from clusters I–VI. (**G**) The control LMH monolayer cells were treated with PBS.

All ARV isolates were propagated in LMH monolayer cells, and the CPE manifested as fusion of numerous cells. Moreover, there were no significant differences in the CPE among the clusters (Figure 2).

### 3.3. Phylogenetic Analysis

**Figure 3:**
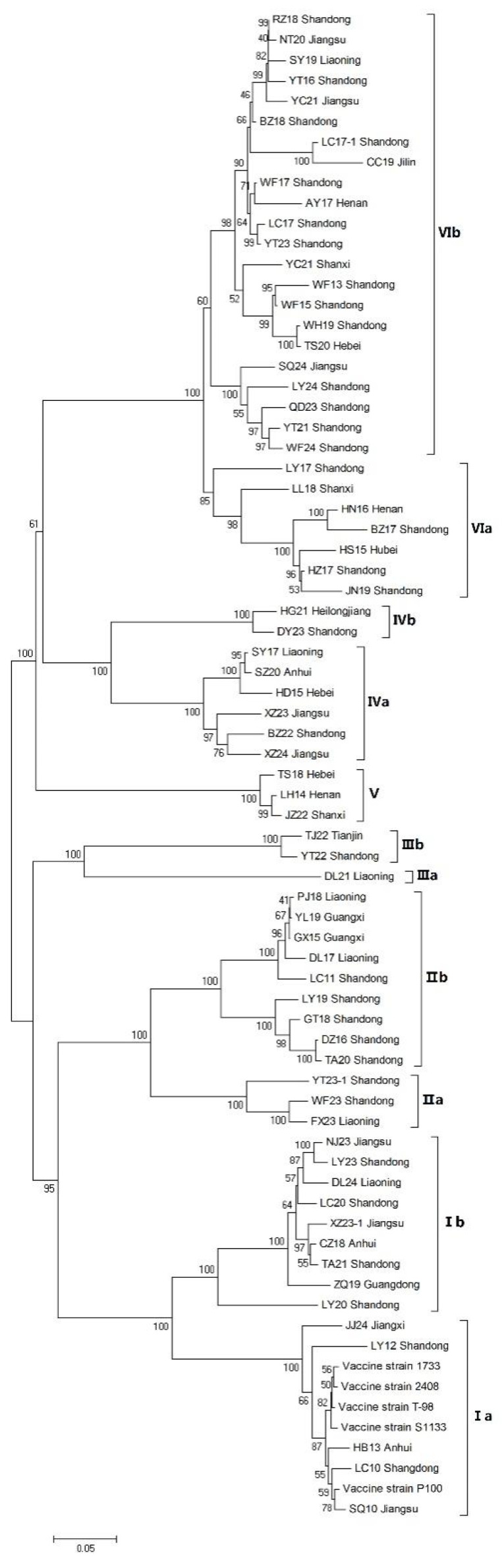
Phylogenetic tree constructed with complete σC gene sequences of the ARV isolates and the vaccine strains using MEGA7.0. Branch lengths are proportional to the evolutionary distances between sequences.

**Figure 4:**
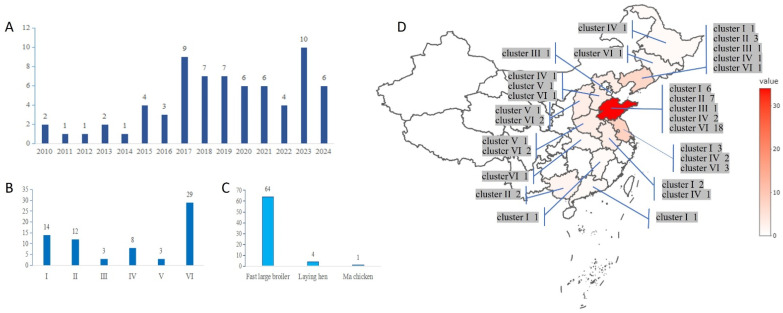
Time, genotype, breed and regional distribution of ARV isolates. (**A**) Time distribution; (**B**) Genotype distribution; (**C**) Breed distribution; (**D**) regional distribution (Numbers represent the number of strains).

The phylogenetic tree was constructed based on the complete σC gene sequence of 69 isolates and the S1133 vaccine strain (Figure 3). These 69 isolates were grouped into 6 clusters and 11 subclusters, including 14 ARV isolates of cluster I, 12 ARV isolates of cluster II, 3 ARV isolates of cluster III, 8 ARV isolates of cluster IV, 3 ARV isolates of cluster V, and 29 ARV isolates of cluster VI. In general, most strains isolated from China belonged to cluster VI. Among the 69 isolates, 64 were from fast large broilers, 4 were from laying hens, and 1 was from a Ma chicken (Figure 4).

### 3.4. Homology Analysis

**Figure 5:**
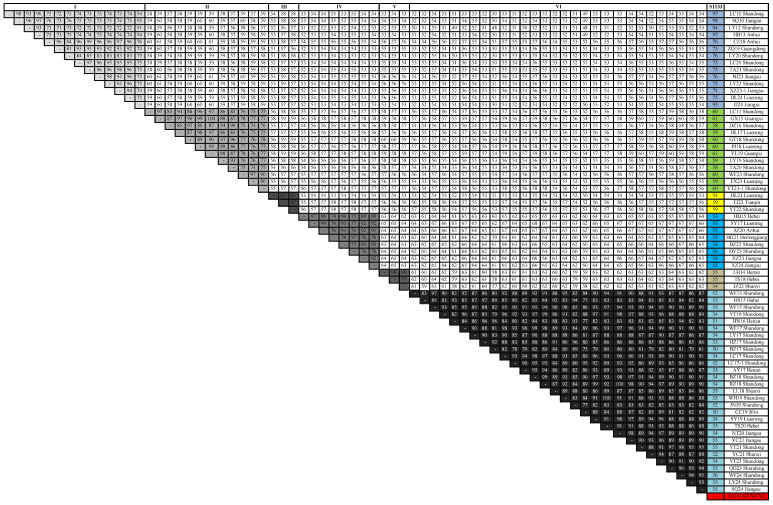
Percentage of identity between the deduced ARV σC gene sequences from the 69 isolates and the S1133 vaccine strain.

The σC gene of 69 ARV isolates was amplified and sequenced followed by alignment and analysis using the MegAlign program. The identity and divergence of nucleotide sequences between the ARV isolates and the S1133 vaccine strain were compared (Figure 5). The highest homology among the ARV isolates was 99.9% and the lowest homology was 48.0%. The sequence identities of the ARV isolates grouped in clusters I, II, III, IV, V, and VI varied from 70.5 to 99.0%, 73.6 to 99.9%, 63.0 to 97.2%, 74.4 to 99.3%, 96.7 to 98.8%, and 74.6 to 99.6%, respectively. The sequence identity between the S1133 vaccine strain and the ARV isolates, except cluster I, ranged from 50.3 to 60.8%. The ARV isolates in cluster I shared 73.4–98.0% homology with the S1133 vaccine strain.

### 3.5. Pathogenicity in SPF Chickens

**Figure 6:**
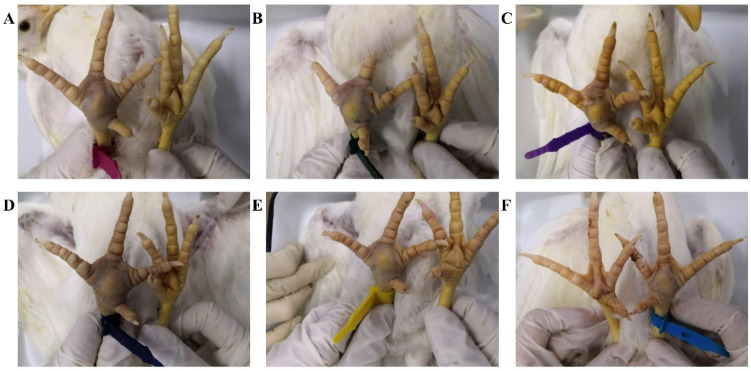
Pathogenicity of ARV isolates in SPF chickens. (**A**–**F**) One footpad inoculated with the ARV isolates from clusters I–VI was swollen. The other footpad, which was inoculated with PBS as a control, showed no significant change.

The pathogenicity experiments in SPF chickens showed that all six ARV isolates from different clusters were able to cause swelling of the footpad with subcutaneous bruise-like purple to reddish discoloration (Figure 6). Except for the PJ18 strain (clusters II), which showed a 90% incidence of footpad lesions, the other five strains showed a 100% incidence of footpad lesions. However, no significant lesions were observed in the viscera. Based on gross lesions, there was no significant difference in the severity of disease induced by different ARV isolates from different clusters.

## 4. Discussion

ARV has become an important pathogen in Chinese poultry, and mainly causes viral arthritis and tenosynovitis. Besides biosecurity, vaccination is the most effective option to prevent this disease. Nevertheless, due to the genetic variability of ARVs, the commercial vaccines used at present do not provide adequate protection against field challenges of variant strains [17,18,19,20,21,23]. Therefore, it is necessary to develop a new approach to preventing and controlling this disease.

Phylogenetic analysis is important not only for the selection of vaccine candidates but also for monitoring ARV antigenic drift after vaccination. The effectiveness of a vaccine largely depends on the antigenic similarity between the vaccine and field strains. Because the σC gene is considered to be the most variable gene and encodes the σC protein responsible for host cell attachment and eliciting neutralizing antibodies [2,24], we focused on the σC gene to investigate molecular phylogeny and analyze homology.

At present, the genotyping standards of ARV are not uniform. In 2017, Ayalew et al. classified 37 ARVs isolated from Canada and 63 ARVs from the NCBI into six clusters, I–VI, based on the σC gene. In this study, we conducted genotype analysis on 69 ARV isolates from China using this typing method. These ARVs were divided into 6 clusters and 11 subclusters. In cluster I, the ARVs in subcluster Ia had high homology with the S1133 vaccine strain (93.5–98.0%), while the ARVs in subcluster Ib had low homology with the S1133 strain (73.4–76.4%). In cluster II, the ARVs isolated in 2023 shared less than 80% homology with other ARVs. Therefore, they were divided into two subclusters. There were only three isolates in cluster III, which were also divided into two subclusters. The DL21 strain had only 63.0–63.4% homology with the other two stains. In cluster IV, the HG21 and DY23 strains had 74.4–76.7% homology with the other six strains, and were divided into a subcluster. The cluster VI viruses, the main epidemic genotype in China, were divided into two subclusters, VIa and VIb, with a homology of 75–90% between these two subclusters. The cluster VI strains had only 50.3–55.7% homology with the S1133 strain. In addition, isolates from cluster II–V had 53.4–60.8% homology with the S1133 strain. The prevalence of ARV variants in China has been reported previously. In 2016, Zhong et al. isolated 11 ARV strains from northern China and classified them into three genotypes, lineages 1–3, based on the variation of the σC gene. In 2019, Zhang et al. conducted genetic evolution analysis on the σC gene of 18 ARV strains isolated from China and classified them into six genotypes. All these results indicated that ARV isolates varied greatly in China and were genetically distant from the vaccine strain.

The LMH cell line is an avian cell line [25] that can be infected by a variety of poultry viruses, including ARV [26,27,28,29]. In the present study, we selected LMH cells for ARV propagation, and all ARV isolates caused cell fusion in culture, which is a prominent feature and convenient for the propagation of ARV.

Establishment of an animal challenge model is necessary to evaluate the pathogenicity of ARVs and the effectiveness of vaccines. Because SPF chickens at 21 days of age have a healthy immune system and are capable of producing antibodies to the inactivated vaccine, we used SPF chickens at 21 days of age for animal experiments. In the present study, footpad injection efficiently caused foot swelling in experimental chickens starting at day three after injection and consistently persisted for more than 20 days, which allowed evaluation of the pathogenicity of ARVs and the effectiveness of vaccines based on the degree of foot swelling.

In conclusion, the present study demonstrated that most avian reovirus strains currently prevalent in China have undergone significant genetic mutations compared to traditional vaccine strains. This has resulted in poor protective effects of vaccines in clinical production. This notion was also confirmed by the results of serological studies [17,30]. Therefore, an update of avian reovirus vaccine strains is urgently needed.

## Figures and Tables

**Table 1:** Detailed results of diagnostic RT-PCR for 409 tissue samples.

Areas	Bird Type					Total No.	No. of Positive Samples	Rate of Positive Samples
	Fast Large Broiler	Laying Hen	817 Broiler	Ma Chicken	Sanhuang Broiler			
Shandong	102	8	13	3	0	126	40	31.75%
Jiangsu	45	5	3	2	0	55	18	32.73%
Henan	17	4	3	1	0	25	4	16.00%
Hebei	28	7	2	1	0	38	8	21.05%
Zhejiang	5	0	0	0	1	6	1	16.67%
Jiangxi	7	0	1	2	0	10	1	10.00%
Anhui	13	1	0	0	0	14	2	14.29%
Hunan	2	0	0	0	1	3	0	0.00%
Hubei	5	0	0	0	1	6	0	0.00%
Fujian	2	0	0	0	1	3	0	0.00%
Guangdong	11	0	0	0	1	12	2	16.67%
Guangxi	16	0	0	0	0	16	4	25.00%
Liaoning	43	1	0	0	0	44	16	36.36%
Jilin	10	0	0	0	0	10	2	20.00%
Heilongjiang	20	0	0	0	0	20	7	35.00%
Tianjin	4	0	0	0	0	4	0	0.00%
Sichuan	2	0	0	0	0	2	1	50.00%
Shanxi	11	3	1	0	0	15	5	33.33%
Total	343	29	23	9	5	409	111	27.14%

## Data Availability

All important data generated during the current study are included in the manuscript. Additional data related to this article may be requested from the corresponding author.
